# A novel neuregulin – jagged1 paracrine loop in breast cancer transendothelial migration

**DOI:** 10.1186/s13058-018-0960-8

**Published:** 2018-04-10

**Authors:** Ramon M. Cabrera, Serena P. H. Mao, Chinmay R. Surve, John S. Condeelis, Jeffrey E. Segall

**Affiliations:** 1 Department of Anatomy and Structural Biology, Price 201, Albert Einstein College of Medicine, 1301 Morris Park Avenue, Bronx, NY 10461 USA; 2 Gruss Lipper Biophotonics Center, Albert Einstein College of Medicine, Bronx, NY 10461 USA; 3 Integrated Imaging Program, Albert Einstein College of Medicine, Bronx, NY 10461 USA

**Keywords:** Intravasation, Neuregulin, Jagged1, Breast cancer, Macrophage

## Abstract

**Background:**

The interaction of breast cancer cells with other cells in the tumor microenvironment plays an important role in metastasis. Invasion and intravasation, two critical steps in the metastatic process, are influenced by these interactions. Macrophages are of particular interest when it comes to studying tumor cell invasiveness. Previous studies have shown that there is paracrine loop signaling between breast cancer cells and macrophages involving colony stimulating factor 1 (CSF-1) produced by tumor cells and epidermal growth factor (EGF) production by macrophages. In this paper, we identify a novel paracrine loop between tumor cells and macrophages involving neuregulin (NRG1) and notch signaling.

**Methods:**

The aim of this study was to determine the role of NRG1, a ligand of the ErbB3 receptor, in macrophage stimulation of tumor cell transendothelial migration and intravasation. We used fluorescence-activated cell sorting (FACS) and western blot to determine ErbB3 and NRG1 expression, respectively. An in vitro transendothelial migration (iTEM) assay was used to examine the effects of short hairpin (sh)RNA targeting NRG1 in tumor cells and clustered regularly interspaced short palindromic repeats (CRISPR) knockout of jagged 1 (JAG1) in macrophages. Orthotopic xenograft injections in mice were used to confirm results in vivo*.*

**Results:**

In our system, macrophages were the primary cells showing expression of ErbB3, and a blocking antibody against ErbB3 resulted in a significant decrease in macrophage-induced transendothelial migration of breast cancer cells. Stimulation of macrophages with NRG1 upregulated mRNA and protein expression of JAG1, a ligand of the Notch receptor, and JAG1 production by macrophages was important for transendothelial migration of tumor cells.

**Conclusions:**

This study demonstrates that stimulation of macrophages by tumor cell NRG1 can enhance transendothelial migration and intravasation. We also demonstrate that this effect is due to induction of macrophage JAG1, an important ligand of the Notch signaling pathway.

## Background

In women, breast cancer is the most commonly diagnosed and second leading cause of cancer death [1]. Although there have been significant improvements in screening techniques and information on prevention, breast cancer mortality occurs due to the metastatic spread of cells from the primary tumor to distant sites. The process of metastasis involves many steps, including invading the basement membrane and normal surrounding breast tissue, entering the bloodstream (intravasating), and then extravasating at distant organs to initiate metastasis formation [2]. Although cancer cells have their own inherent invasive properties, interaction with other cell types in the tumor microenvironment can facilitate metastasis. Specifically, tumor associated macrophages (TAMs) can play a role in breast cancer metastasis, and higher TAM density has been associated with worse prognosis [3, 4]. In particular a subset of TAMs form the tumor microenvironment of metastasis (TMEM), the doorway for intravasation in breast tumors, which is directly involved in systemic tumor cell dissemination [5]. TMEM is a clinically validated prognostic marker of metastatic risk in patients with breast cancer [6, 7]. Previous work has shown paracrine loop signaling between tumor cells and macrophages, where epidermal growth factor (EGF) from macrophages induced by colony stimulating factor 1 (CSF-1) from tumor cells contributes to breast cancer cell motility and invasion [8, 9]. Recent work has also indicated that this paracrine signaling is important to sensitize tumor cells to hepatocyte growth factor (HGF) signaling by endothelial cells to attract them towards blood vessels [10].

In examining the relationship between tumor cells and macrophages, we sought to identify additional signaling pathways that may be working in conjunction with the canonical paracrine loop to contribute to breast cancer metastasis. Studies characterizing macrophages co-invading with breast cancer cells showed higher expression of ErbB3, a member of the epidermal growth factor receptor (EGFR) family, in the invasive macrophages [11, 12]. This along with data showing that expression of ErbB3 in solid tumors is associated with worse prognosis [13] made it an attractive target for further exploration. ErbB3 has been widely studied in cancer cells, but little is known about its role in macrophages. Utilizing both in vitro and in vivo techniques, we wanted to determine the role of ErbB3 and its ligand neuregulin1 (NRG1) in tumor cell intravasation.

In the studies reported here, we identified a novel paracrine loop for intravasation, in which NRG1 production by tumor cells stimulates macrophages to produce JAG1, resulting in increased transendothelial migration.

## Methods

### Cell culture and generation of stable cell lines

Breast cancer cell lines BT549 and MDA-MB 231 were cultured in Dulbecco’s modified Eagle medium (DMEM) (cat# SH30243, Hyclone, GE Healthcare Life Sciences, Logan, UT, USA) supplemented with 10% fetal bovine serum (FBS) (cat# S11550, Altanta Biologicals, Flowery Branch, GA, USA). BAC 1.2F5 macrophages [14] were cultured in Minimum Essential Medium, Alpha (α-MEM) (cat# 15-012-CV, Corning, Tewksbury, MA, USA) supplemented with 10% fetal bovine serum (cat# 100-106, Gemini Bio-Products, Sacramento, CA, USA) and 3000 U/mL CSF-1 (a gift from Chiron Corp, Emeryville, CA, USA). Human umbilical vein endothelial cells (HUVEC) were cultured in EGM-2 medium (cat# CC-3162, Lonza, Allendale, NJ, USA) according to the manufacturer’s instructions and not used beyond passage 4 for any experiments.

The pTRIPZ empty vector or constructs containing NRG1 shRNA (sequences in Table 1) (Dharmacon, Lafayette, CO, USA) were transfected into 293 cells along with the TAT, REV, GAG/POL packaging plasmids and VSV-g pseudotyping plasmid using Lipofectamine 2000 (cat# 11668019, Invitrogen, Carlsbad, CA, USA). MDA-MB 231 cells were then transduced using supernatants containing virus and 8 μg/mL polybrene (TR-1003-G, EMD Millipore, Billerica, MA, USA). Stable transductants were identified by placing the cells under puromycin selection (1 μg/mL) for 1 week. Cells were grown in the presence of 2 μg/mL doxycycline for at least 5 days to induce shRNA expression. For the rescue experiments, cells containing the pTRIPz vector or NRG1 shRNA1 were transfected with either a control vector (VB160226-10013) or NRG1 expression vector (VB160118-10047) both from VectorBuilder (Cyagen Biosciences Inc., Santa Clara, CA, USA).

**Table 1:** Neuregulin (NRG1) short hairpin RNA (shRNA) sequences

NRG1 shRNA vector	shRNA sequence
shRNA1	TCTTGAACCACTTGAATCT
shRNA2	TAGATCTGGTAAGACACAT

For the knockout of Jagged 1 in the BAC1.2F5 cells, either a control non-targeted or JAG1 targeted guide RNA (gRNA) (sequences in Table 2) was inserted into the lentiCRISPR v2 construct (Addgene plasmid #52961) and then transformed into Stbl3 bacteria. DNA was then isolated and transfected into 293 cells as described above. Virus-containing supernatants were collected and used to transduce macrophages. Cells were then placed under puromycin selection (2 μg/mL) and western blot was used to screen for gene deletion.

**Table 2:** Jagged 1 (JAG1) clustered regularly interspaced short palindromic repeats (CRISPR) guide sequences

Target	Sequence
Non-targeting guide	GCACTACCAGAGCTAACTCA
JAG1-specific guide	CAGTCCCGCGTCACTGCCGG

### Western blot

Cells were washed once with phosphate-buffered saline (PBS), lysed using sample buffer containing 10% SDS and analyzed by SDS-PAGE. Membranes were imaged on a Li-Cor scanner, and processed using ImageJ. To examine the induction of JAG1 protein expression, BAC cells were stimulated with 12 nM NRG1 (cat# 396-HB, R&D Systems, Minneapolis, MN, USA) for 8 h and lysed: antibodies and their dilutions were used as follows: Tubulin (1:5000) (cat# T4026, Sigma-Aldrich, St. Louis, MO, USA), NRG1 (1:1000) (cat# sc-28,916, Santa Cruz Biotechnology, Dallas, TX, USA), Jagged 1 (1:1000) (cat# sc-6011, Santa Cruz Biotechnology).

### Fluorescence-activated cell sorting (FACS)

Cells were detached with 2 mM EDTA, centrifuged, and resuspended at a concentration of 5 × 10^6^ cells per mL in 100 μL in a FACS buffer containing PBS, 2 mM EDTA, and 2.5% FBS. Cells were then placed on ice and treated for 5 min with 5 μg/mL Fc Block (cat# 553142, BD Biosciences, San Jose, CA, USA). Then, either the ErbB3 blocking antibody (cat# MS-303-PABX, Thermo Scientific, Fremont, CA, USA) or mouse IgG1 isotype control (cat# 0102-01 Southern Biotech, Birmingham, AL, USA) were added at a concentration of 10 μg/mL for 30 min, with mixing of the tubes by flicking every 10 min to ensure proper labeling. Samples were then centrifuged and washed three times in the FACS buffer to eliminate any unbound antibody. Cells were then labeled with a donkey anti-mouse Alexa-647 conjugated secondary antibody (cat# 715-605-151, Jackson Immunoresearch, West Grove, PA, USA) for 30 min. Samples were then washed and filtered in preparation for FACS analysis. A total of 1 × 10^4^ cells per sample were analyzed using a DXP10 Calibur flow cytometer and sample data were processed using FlowJo.

### Quantitative real-time polymerase chain reaction (qRT-PCR)

To quantify gene expression, cells were grown under normal culture conditions. Total RNA was isolated using the RNeasy mini kit (cat# 74134, Qiagen, Germantown, MD, USA) and complementary (c)DNA was synthesized and amplified from total RNA using the Superscript II system (cat# 11904-018, Thermofisher Scientific, Fremont, CA, USA). Baseline expression was measured using a SYBR Green Mastermix and gene-specific primers (sequences in Table 3). For the co-culture assay, gene-specific probes and primers from the Taqman system were used to perform real-time PCR, with each reaction being done in triplicate (Human JAG1 cat# Hs00164982_m1, Mouse JAG1 cat# Mm00496902_m1, Human glyceraldehyde-3-phosphate dehydrogenase (GAPDH) cat# Hs02758991_g1, Mouse GAPDH cat# Mm99999915_g1, Thermofisher Scientific, Fremont, CA, USA). The mean cycle threshold (Ct) values were then used to analyze relative expression. Analysis was done using the ΔΔCt method in which all Ct values were normalized to GAPDH.

**Table 3:** Gene-specific primer sequences

Gene	Sequence
Mouse NRG1	F: ATAAAGTGTCGCGAGAAGGAG R: GTAGTTTTGGCAACGATCACC
Human NRG1	F: CACTATACTTCCACAGCCCATC R: TGTGCCTACTGTTTTCTACGG
Mouse GAPDH	F: CTGGAGAAACCTGCCAAGTA R: TGTTGCTGTAGCCGTATTCA
Human GAPDH	F: ACATCGCTCAGACACCATG R: TGTAGTTGAGGTCAATGAAGGG

*NRG1* neuregulin, *GAPDH* glyceraldehyde-3-phosphate dehydrogenase

### In vitro transendothelial migration assay (iTEM)

The iTEM assay was performed as previously described [15]. Briefly, transwells from EMD Millipore (cat# MCEP24H48) were coated with 2.5 μg/mL Matrigel (cat# 356230, BD Biosciences, San Jose, CA, USA) in a total volume of 50 μL. Then approximately 1 × 10^4^ human umbilical vein endothelial cells in 50 μL of EGM-2 medium were plated on the inverted transwells previously coated with Matrigel and allowed to adhere for 4 h at 37 °C. Transwells were then placed into a 24-well plate with 1 mL of EGM-2 in the bottom well and 200 μL inside the upper chamber and allowed to grow for 48 h in order to form a monolayer. Breast cancer cells were labeled with cell tracker green dye and macrophages with cell tracker red (Green cat# C7025, Red cat# C34552, Invitrogen, Carlsbad, CA, USA), resuspended in M199 media (cat# SH30253.01, Hyclone) and plated at 15,000 breast cancer cells and 60,000 macrophages per transwell and allowed to transmigrate towards EGM-2 containing 3000 U/mL CSF-1 for 18 h. For treatment with JAG1 or scrambled peptide, tumor cells were serum starved overnight in DMEM and then pre-incubated with 30 uM of either Jagged 1 DSL peptide (AS-61298, AnaSpec) or Jagged 1 Scrambled peptide (AS-64239, AnaSpec) in serum starvation medium for 4 h at 37 °C before labeling and plating in the transwell. Samples were then fixed in 4% paraformaldehyde, permeabilized with 0.1% Triton-X 100 and stained with rhodamine phalloidin (cat# R415, Invitrogen). Transwell membranes were excised and mounted, with Z-series taken in eight random fields per sample.

### Animal studies

All *in vivo* experiments were conducted in accordance with the National Institutes of Health regulations on the care and use of experimental animals and approved by the Albert Einstein College of Medicine Animal Use Committee. Orthotopic tumor xenografts were generated by injecting a total of 2 × 10^6^ MDA-MB 231 cells suspended in sterile PBS with 20% Collagen I (cat# 354249, Corning, Corning, NY, USA) into the inguinal (4th from top) right mammary fat pad of 5-week-old to 8-week-old female mice with severe combined immunodeficiency (SCID) (NCI). Peripheral blood, primary tumors, and lungs were collected when the tumors reached approximately 1 cm in diameter.

Circulating tumor cells were collected by anesthetizing mice and drawing blood from the right atrium using syringes containing 50 μL of heparin to prevent clotting during collection: 500 μL to 1 mL of blood was collected per mouse. Blood was then placed in 9 mL of 1 × red blood cell lysis buffer for 10 min, centrifuged, and resuspended in 10 mL DMEM/F12 medium in a 10-cm cell culture dish. After 3 days of culture, growth medium was changed to DMEM/F12 containing doxycycline to induce red fluorescent protein (RFP) for tumor cell counting (doxycycline treatment did not affect cell growth). A week after collection, samples were counted under a fluorescence microscope, using turbo RFP expression to identify tumor cells. Intravasation was calculated by dividing the number of colonies per plate by the volume of blood collected and normalizing to 1 mL.

### Statistical analysis

Results are representative of at least three independent experiments for in vitro experiments and at least 11 mice per group in in vivo experiments. Statistical analysis was performed using the unpaired or paired two-tailed Student’s *t* test, or *z* test as indicated.

## Results

### ErbB3 is expressed on macrophages and NRG1 protein is expressed by tumor cells

**Fig. 1:**
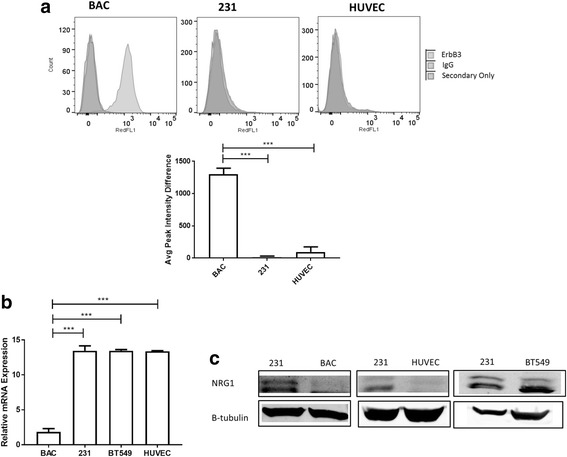
ErbB3 is expressed on macrophages and neuregulin (NRG1) is expressed by tumor cells. **a** BAC macrophages, MDA-MB 231 breast cancer cells (231), and human umbilical vein endothelial cells (HUVEC) were analyzed using fluorescence-activated cell sorting (top) to determine surface ErbB3 expression. Cells were labeled with an ErbB3 blocking antibody reactive to both mouse and human ErbB3, with the corresponding IgG isotype and secondary antibody only as the experimental controls. Quantitation (bottom) is shown as the mean and SEM for the ErbB3 blocking antibody minus the mean signal of the isotype control from three independent experiments: ***p < 0.001, *t* test. **b** Quantitation of NRG1 mRNA expression by qRT-PCR. Values calculated as: (20-Delta cycle threshold (CT)). N = 3; ***p < 0.001, *t* test. **c** Detection of NRG1 protein expression by western blot, showing a doublet of bands at 32–34 kiloDalton (kDa) most strongly in MDA-MB 231 and BT549 cells

In order to determine surface expression levels of ErbB3, macrophages (BAC), MDA-MB 231 breast cancer cells (231), and endothelial cells (HUVEC) were labeled with an ErbB3 blocking antibody and analyzed by FACS. Of the three cell types, only the macrophages showed significant ErbB3 surface expression (Fig. 1a). After establishing expression of ErbB3 on the macrophages, we then determined expression of the ErbB3 ligand NRG1 in the same cell lines. Using qRT-PCR, we saw that there was very little NRG1 messenger (m)RNA expression in the macrophages, while high levels of mRNA were present in the tumor cells and endothelial cells (Fig. 1b). NRG1 protein expression was evaluated in the three different cell types: western blot analysis of cell lysates showed strong NRG1 protein expression in the MDA-MB 231 cells, and a second breast cancer triple negative cell line BT549, showed less expression in the HUVECs and macrophages (Fig. 1c). In summary, NRG1 was most highly expressed by the tumor cells, while the receptor ErbB3 was mainly present on the surface of macrophages.

### Blocking ErbB3 reduces macrophage-induced transendothelial migration (iTEM)

**Fig. 2:**
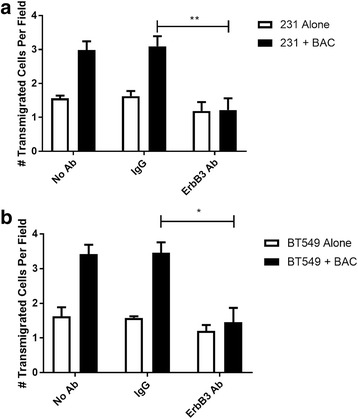
Blocking ErbB3 inhibits macrophage-induced transendothelial migration**. a** MDA-MB 231 cells were placed in transwells coated with Matrigel and endothelial cells, either alone (231 Alone) or in the presence of macrophages (231 + BAC), and left untreated (No Ab) or with either an IgG isotype control (IgG) or ErbB3 blocking antibody (ErbB3). Transendothelial migration was measured after 24 h. Results are shown as the mean and SEM of the number of tumor cells crossing the endothelial layer per field. N = 4; **p < 0.01, *t* test. **b** BT549 cells were placed in the transendothelial migration (iTEM) assay either alone (BT549 Alone) or with macrophages (BT549 + BAC) either untreated, with a control IgG, or with an ErbB3 blocking antibody. Results are shown as the mean and SEM of the number of tumor cells crossing the endothelial layer per field. N = 3; *p < 0.05, *t* test

To examine the effects of blocking ErbB3 signaling on intravasation, tumor cells and macrophages were placed in a previously established in vitro iTEM assay [15]. This assay models the conditions seen at the interface of the tumor and the blood vessel and allows us to quantify the number of tumor cells that are crossing the endothelial cell layer. It has previously been seen that although breast cancer cells are able to cross the endothelial cell barrier, transendothelial migration is enhanced in the presence of macrophages as shown in Fig. 2 and in previous publications [15, 16]. In the untreated and IgG control conditions the tumor cells alone exhibited a basal level of transendothelial migration, which increased in the presence of macrophages. However, in the presence of an ErbB3 blocking antibody, there was no enhancement of transendothelial migration in the presence of macrophages (Fig. 2a). Similar results were found for the BT549 cells when placed in the iTEM assay (Fig. 2b). Thus inhibition of ErbB3 on macrophages blocked the ability of macrophages to enhance tumor cell transmigration.

### Knockdown of NRG1 in tumor cells reduces macrophage-induced transendothelial migration in vitro

**Fig. 3:**
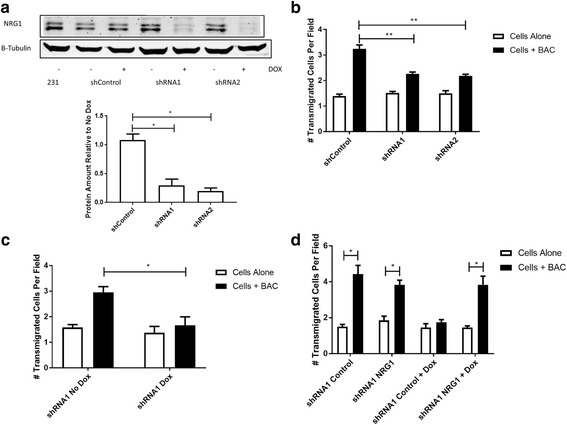
Knockdown of neuregulin (NRG1) reduces macrophage-induced transendothelial migration. **a** Western blot (top) showing knockdown of NRG1 protein expression in MDA-MB 231 by NRG1-targeted shRNA constructs(shRNA1, shRNA2) but not by empty vector control (shControl) after culture in the presence of doxycycline (+) compared to culture in the absence of doxycycline (−) or parental (231). Quantitation of knockdown (bottom) shown as the relative expression of NRG1 in the various lines with versus without doxycycline. Results presented as mean and SEM of NRG1 protein expression in doxycycline-treated cells divided by expression in untreated cells in three independent experiments; *p < 0.05, *t* test. **b** Cells containing the vector control or shRNA constructs were treated with doxycycline for 5 days and assayed for transendothelial migration after 24 h in the absence or presence of macrophages. Data are means and SEM from three independent experiments; **p < 0.01, *t* test. **c** Transendothelial migration of cells containing the shRNA1 construct but not induced (No Dox) was compared to doxycycline-treated cells (Dox). Data are means and SEM from three independent experiments; *p < 0.05, *t* test. **d** shRNA1 cells transduced with empty rescue vector control (Control) or vector containing NRG1 rescue construct (NRG1) were cultured in the absence or presence of doxycycline (Dox). Treatment with doxycycline blocked the macrophage enhancement of in vitro transendothelial migration (iTEM) in the control vector line, but expression of the NRG1 construct rescued macrophage enhancement. Data are means and SEM from three independent experiments; *p < 0.05 by *t* test

After establishing that ErbB3 signaling on macrophages was important for enhancing breast cancer cell migration across the endothelial cell layer, we then tested whether altering expression of the ErbB3 ligand NRG1 by the cancer cells was important. In order to knock down expression of NRG1 in the MDA-MB 231 breast cancer cell line, we used the TRIPZ doxycycline-inducible vector [17] containing either short hairpin (sh)RNAs targeting the NRG1 gene or a control empty vector. In addition to driving shRNA expression, the vector also contains an RFP construct, which leads to fluorescence when the shRNA is expressed. After growing the transduced cell lines in the presence of doxycycline and checking for the expression of RFP, we were able to detect knockdown of NRG1 protein in western blot by two different shRNA sequences (Fig. 3a). After confirming NRG1 knockdown, we then placed the shRNA expressing cells into the iTEM assay to observe the effect of reducing NRG1 expression on transendothelial migration. The cells containing the control vector showed the expected increase in crossing the endothelial cell layer when macrophages were added, but this effect was significantly reduced for the NRG1 shRNA-expressing tumor cells (Fig. 3b). This reduction in transendothelial migration was specifically due to the induction of shRNA expression, since cells carrying the NRG1 shRNA vector but grown without doxycycline still showed macrophage-induced transendothelial migration, compared to cells grown in parallel where the shRNA expression was induced (Fig. 3c). To confirm that the reduction in transendothelial migration seen in the cells expressing NRG1 shRNA was due to loss of NRG1, an NRG1 construct resistant to the shRNA was designed. TRIPZ control and NRG1 shRNA cells were transfected with the rescue NRG1 expression vector or empty vector control and tested in the iTEM assay. Even after induction of the NRG1 shRNA, cells expressing the NRG1 rescue construct showed increased transendothelial migration in the presence of macrophages (Fig. 3d), confirming the role of NRG1.

### Knockdown of NRG1 in tumor cells reduces intravasation in vivo

**Fig. 4:**
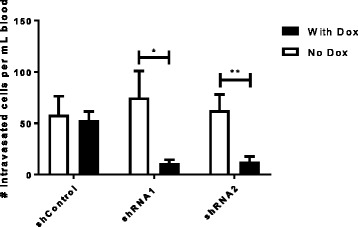
Neuregulin (NRG1) knockdown reduces intravasation in vivo*.* Intravasation was measured in mice bearing orthotopic tumors carrying the control vector (shControl) or NRG1 shRNA constructs (shRNA1, shRNA2). Mice were fed with control (no dox) or diet containing doxycycline (with dox) to induce tumor expression of shRNA. Results are shown as the average number of tumor cells per 1 mL of blood. Data are means and SEM from least 11 animals per group; **p < 0.01,*p < 0.05, *t* test

As a means to validate the results observed in our in vitro studies, we used a xenograft mouse model to study the effects of NRG1 expression on intravasation. MDA-MB 231 cells expressing either the control vector or NRG1 shRNA vectors were orthotopically injected into the mammary fat pads of SCID mice. The mice were then fed with either a control diet or with food containing doxycycline (inducing the shRNA). When tumors reached approximately 1 cm in diameter, peripheral blood was collected from the right atrium. Doxycycline treatment did not affect the growth rate of tumors, but there was a significant decrease in the number of circulating tumor cells in mice containing tumors with the NRG1 shRNA construct, which were fed the diet containing doxycycline (Fig. 4). Rates of metastasis to the lung were relatively low and we did not detect a statistically significant difference in metastasis (data not shown).

### Jagged 1 (JAG1) is upregulated in NRG1-stimulated macrophages and is important for tumor cell transendothelial migration

**Fig. 5:**
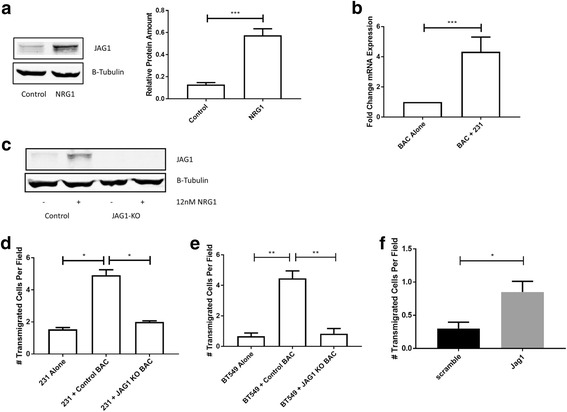
Neuregulin (NRG1) induces jagged 1 (JAG1) expression in macrophages and is important for in vitro transendothelial migration (iTEM). **a** Western blot (left) showing induction of JAG1 protein expression in BAC after NRG1 stimulation. Quantitation of protein level is shown on the right. Data are means and SEM from three independent experiments; *p < 0.001, *t* test. **b** Quantitation of JAG1 mRNA induction in BACs after co-culture with MDA-MB 231. Values expressed as fold change of co-culture versus BAC alone. Data are means and SEM trom three independent experiments; **p < 0.001 by *z* test. **c** Western blot of clustered regularly interspaced short palindromic repeats (CRISPR) knockout of JAG1 in BAC cells showing lack of JAG1 expression compared to control guide RNA (gRNA) in the presence or absence of NRG1. **d** Transendothelial migration of MDA-MB 231 cells alone, with control gRNA BACs, or JAG1 gRNA knockout BACs, confirming the importance of macrophage JAG1 in intravasation. Data are means and SEM from three independent experiments; *p < 0.05, *t* test. **e** Transendothelial migration of BT549 cells alone, with control gRNA BACs, or JAG1 gRNA knockout BACs, further confirming the importance of macrophage JAG1 in intravasation. Data are means and SEM from three independent experiments; **p < 0.01, *t* test. **f** Transendothelial migration of MDA-MB 231 cells in the presence of a scrambled control peptide (scramble) or JAG1 peptide (Jag1). Data are means and SEM from three independent experiments; *p < 0.05, paired *t* test

To further elucidate the mechanism of macrophage-enhanced transendothelial migration, we examined the effects of NRG1 stimulation on macrophages. We found that treating macrophages with NRG1 led to increased expression of JAG1, a ligand of the Notch signaling pathway. We saw an increase in JAG1 mRNA (not shown) and protein (Fig. 5a). mRNA levels of the other Notch ligands were not increased (data not shown). We were also able to detect an increase in macrophage (mouse) JAG1 mRNA expression when human MDA-MB 231 breast cancer cells were co-cultured with macrophages (Fig. 5b). It has previously been seen that the Notch receptor on breast cancer cells plays an important role in invasion and intravasation in the iTEM assay [16]; therefore we tested whether altering expression of JAG1 in macrophages had an effect on tumor cell transendothelial migration. To achieve this, we used CRISPR to knock out JAG1 in macrophages (Fig. 5c). We then evaluated our clustered regularly interspaced short palindromic repeats (CRISPR) JAG1 knockout (KO) macrophages in the iTEM assay. In comparison to the macrophages transduced with a control non-targeting guide RNA, we found that the macrophages lacking JAG1 expression did not enhance transendothelial migration of MDA-MB 231 cells, indicating an important role for macrophage JAG1 expression (Fig. 5d). This was confirmed with a second breast cancer cell line, BT549, which also showed no enhancement of transendothelial migration when placed in an iTEM assay with macrophages lacking JAG1 expression (Fig. 5e**)**. Addition of an exogenous JAG1 peptide alone can significantly increase transendothelial migration of MDA-MB 231 cells, supporting the conclusion that stimulation of the Notch receptor on tumor cells by JAG1 can enhance intravasation (Fig. 5f**)**.

## Discussion

**Fig. 6:**
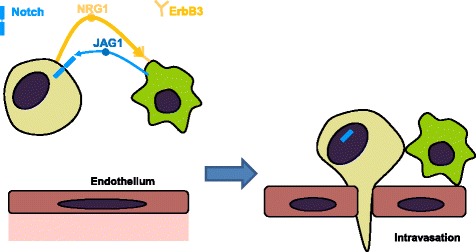
Proposed model. Neuregulin **(**NRG1) produced by tumor cells binds to ErbB3 on macrophages, stimulating production of the Notch ligand jagged 1 (JAG1). JAG1 in turn binds to Notch on tumor cells, stimulating intravasation

Our results provide new insights into the interaction between breast cancer cells and macrophages and how signaling between the two cell types can contribute to breast cancer intravasation. Using the in vitro transendothelial migration assay (iTEM), we found that an ErbB3 blocking antibody inhibited macrophage stimulated iTEM. Since the major source of the ErbB3 ligand NRG1 in the cell types we used was the tumor cells, we evaluated the effect of suppressing NRG1 expression in tumor cells. We found that reducing NRG1 in the tumor cells reduced the ability of macrophages to stimulate iTEM, consistent with a model where NRG1 produced by tumor cells stimulates macrophages through ErbB3. We confirmed the importance of NRG1 through rescue with a NRG1 expression construct. Injecting the tumor cells into the mammary fat pads of mice to form a primary tumor indicated that suppression of NRG1 expression also reduced intravasation in vivo. We found that stimulating macrophages with NRG1 induced the production of JAG1, and that knockout of JAG1 in macrophages led to an inhibition of macrophage-stimulated iTEM. This led to a model for macrophage-enhanced iTEM in which NRG1 produced by tumor cells binds to ErbB3 on macrophages and induces expression of JAG1, which in turn enhances tumor cell intravasation (Fig. 6).

Previous work has revealed the importance of breast tumor cell contact with macrophages in TMEM during intravasation [5] and Notch signaling in macrophage-dependent transendothelial migration and intravasation [15, 16]. Those studies demonstrated a role for macrophages in the stimulation of intravasation of tumor cells involving NOTCH on tumor cells. The work presented here is complementary, identifying JAG1 from macrophages as a NOTCH ligand important for the macrophage-stimulated transendothelial migration. For macrophages, JAG1 expression has been well-documented [18, 19]. JAG1 can be induced in macrophages by a variety of stimuli, including growth factors [20], toll-like receptor (TLR) ligands such as lipopolysaccharide (LPS) [21–24], or hypoxia [25]. In our studies, we have found that NRG1 stimulation of macrophages can lead to increased expression of JAG1. The use of an ErbB3 blocking antibody identified ErbB3 as the receptor for NRG1 on macrophages. ErbB4, the other potential receptor for NRG1, was not detectable by PCR, western blot or FACS (data not shown). ErbB3 expression by macrophages, monocytes, and microglia has been previously reported [26–29], possibly involved in suppressing inflammatory responses via NRG1 stimulation. To the best of our knowledge, this is the first report of induction of JAG1 expression by NRG1 in any cell type, including macrophages.

Macrophage-expressed JAG1 has been reported to have a variety of effects. It can mediate juxtacrine effects on T cells [30–33]. In addition, macrophages themselves express Notch receptors and can be altered in polarization [34–36] by JAG1, possibly by autocrine or juxtacrine stimulation. Because Notch signaling plays a role in angiogenesis [37], there is also the possibility that macrophage JAG1 may affect endothelial cells directly, affecting cell junctions and blood vessel permeability, potentially allowing tumor cells to enter the bloodstream. With respect to intravasation, macrophages can stimulate the Notch receptor on the tumor cells, causing them to become more invasive, and increasing their capacity to intravasate [15, 16]. Our work would indicate that JAG1 can mediate this effect. In colorectal cancer, DLL4 expression by tumor-associated macrophages has also been reported [38]. In addition to effects on invasion and intravasation, activation of NOTCH on tumor cells has the potential to enhance stemness and resistance to therapy [39–41]. Interestingly, previous work in melanoma has shown that stimulation of the Notch receptor leads to upregulation of NRG1 [42], and thus it is possible that a positive feedback loop is being activated.

While working with cancer cells in vitro and animal models in vivo serve as valuable tools for studying the behavior of tumor cells and the mechanisms of metastasis, there are a number of limitations that must be considered when analyzing the results and overall impact of our study. Our experiments focused mainly on MDA-MB 231 and BT549 cells, which have been thoroughly characterized. These cells have high NRG1 expression and low surface ErbB3 expression, making autocrine signaling less likely to be a factor in our experiments, even though it has been seen that ErbB3 upregulation may occur in drug-resistant tumors [43]. There are also limitations in the design of our in vitro transendothelial migration assay. While components meant to mimic the extracellular matrix and endothelial cell layer are present, factors such as pressure from fluid flow through vessels, and other cell types, such as fibroblasts, present in the microenvironment are not considered. Our in vitro experiments were performed using mouse macrophages and in vivo experiments with SCID mice. While they do provide a consistent model for what we predict is happening in their human counterparts, differences in species between tumor cells and macrophages must be taken into account. Further experiments to test this model could include macrophage-specific deletion of ErbB3 and JAG1.

Taken as a whole, our data suggest that NRG1 in tumor cells, and ErbB3 and JAG1 in macrophages can play an important role in the metastatic cascade. By inhibiting this signaling pathway between tumor cells and macrophages, intravasation of tumor cells is decreased. A recently published study has shown that conventional chemotherapy may induce metastatic spread [7], leading to worse outcome. Therapies that block cancer cell intravasation could lessen this effect, and also increase the effectiveness of localized treatments such as radiation and surgical resection. Another alternative could be using NRG, ErbB3, JAG1, or Notch as potential biomarkers for metastasis. Past studies have focused on characterizing ErbB3 expression of tumor cells, but our experiments suggest that JAG1 and ErbB3 expression on macrophages may also play an important role.

## Conclusion

These studies demonstrate the potential importance of NRG1 expression by tumor cells in macrophage-enhanced transendothelial migration. We show that NRG1 can stimulate the ErbB3 receptor on macrophages in order to induce JAG1 expression, which acts as a ligand for the tumor cell Notch receptor, increasing their capacity for transendothelial migration and intravasation.

## References

[1] Siegel RL, Miller KD, Jemal A (2017). Cancer Statistics, 2017. CA Cancer J Clin.

[2] Langley RR, Fidler IJ (2011). The seed and soil hypothesis revisited - the role of tumor-stroma interactions in metastasis to different organs. International journal of cancer Journal international du cancer.

[3] Lewis CE, Pollard JW (2006). Distinct role of macrophages in different tumor microenvironments. Cancer Res.

[4] Tsutsui S, Yasuda K, Suzuki K, Tahara K, Higashi H, Era S (2005). Macrophage infiltration and its prognostic implications in breast cancer: the relationship with VEGF expression and microvessel density. Oncol Rep.

[5] Harney AS, Arwert EN, Entenberg D, Wang Y, Guo P, Qian BZ, Oktay MH, Pollard JW, Jones JG, Condeelis JS (2015). Real-time imaging reveals local, transient vascular permeability, and tumor cell intravasation stimulated by TIE2hi macrophage-derived VEGFA. Cancer Discov.

[6] Rohan TE, Xue X, Lin HM, D'Alfonso TM, Ginter PS, Oktay MH, Robinson BD, Ginsberg M, Gertler FB, Glass AG, et al. Tumor microenvironment of metastasis and risk of distant metastasis of breast cancer. J Natl Cancer Inst. 2014;106(8). 10.1093/jnci/dju136. 10.1093/jnci/dju136 PMC4133559 24895374

[7] Karagiannis GS, Pastoriza JM, Wang Y, Harney AS, Entenberg D, Pignatelli J, Sharma VP, Xue EA, Cheng E, D'Alfonso TM, et al. Neoadjuvant chemotherapy induces breast cancer metastasis through a TMEM-mediated mechanism. Sci Transl Med. 2017;9(397). 10.1126/scitranslmed.aan0026. 10.1126/scitranslmed.aan0026 PMC5592784 28679654

[8] Wyckoff J, Wang W, Lin EY, Wang Y, Pixley F, Stanley ER, Graf T, Pollard JW, Segall J, Condeelis J (2004). A paracrine loop between tumor cells and macrophages is required for tumor cell migration in mammary tumors. Cancer Res.

[9] Goswami S, Sahai E, Wyckoff JB, Cammer M, Cox D, Pixley FJ, Stanley ER, Segall JE, Condeelis JS (2005). Macrophages promote the invasion of breast carcinoma cells via a colony-stimulating factor-1/epidermal growth factor paracrine loop. Cancer Res.

[10] Leung E, Xue A, Wang Y, Rougerie P, Sharma VP, Eddy R, Cox D, Condeelis J (2017). Blood vessel endothelium-directed tumor cell streaming in breast tumors requires the HGF/C-Met signaling pathway. Oncogene.

[11] Ojalvo LS, King W, Cox D, Pollard JW (2009). High-density gene expression analysis of tumor-associated macrophages from mouse mammary tumors. Am J Pathol.

[12] Ojalvo LS, Whittaker CA, Condeelis JS, Pollard JW. Gene expression analysis of macrophages that facilitate tumor invasion supports a role for Wnt-signaling in mediating their activity in primary mammary tumors. J immunol (Baltimore, Md : 1950). 2010;184(2):702–12. 10.4049/jimmunol.0902360 PMC3226722 20018620

[13] Ocana A, Vera-Badillo F, Seruga B, Templeton A, Pandiella A, Amir E (2013). HER3 overexpression and survival in solid tumors: a meta-analysis. J Natl Cancer Inst.

[14] Morgan C, Pollard JW, Stanley ER (1987). Isolation and characterization of a cloned growth factor dependent macrophage cell line, BAC1.2F5. J Cell Physiol.

[15] Roh-Johnson M, Bravo-Cordero JJ, Patsialou A, Sharma VP, Guo P, Liu H, Hodgson L, Condeelis J (2014). Macrophage contact induces RhoA GTPase signaling to trigger tumor cell intravasation. Oncogene.

[16] Pignatelli J, Bravo-Cordero JJ, Roh-Johnson M, Gandhi SJ, Wang Y, Chen X, Eddy RJ, Xue A, Singer RH, Hodgson L (2016). Macrophage-dependent tumor cell transendothelial migration is mediated by Notch1/MenaINV-initiated invadopodium formation. Sci Rep.

[17] TRIPZ Inducible Lentiviral shRNA. http://dharmacon.horizondiscovery.com/rnai/shrna/tripz-lentiviral-shrna/. Accessed 6 Apr 2018.

[18] Singh N, Phillips RA, Iscove NN, Egan SE (2000). Expression of notch receptors, notch ligands, and fringe genes in hematopoiesis. Exp Hematol.

[19] Yamaguchi E, Chiba S, Kumano K, Kunisato A, Takahashi T, Takahashi T, Hirai H (2002). Expression of Notch ligands, Jagged1, 2 and Delta1 in antigen presenting cells in mice. Immunol Lett.

[20] Nomaguchi K, Suzu S, Yamada M, Hayasawa H, Motoyoshi K (2001). Expression of Jagged1 gene in macrophages and its regulation by hematopoietic growth factors. Exp Hematol.

[21] Goh F, Irvine KM, Lovelace E, Donnelly S, Jones MK, Brion K, Hume DA, Kotze AC, Dalton JP, Ingham A (2009). Selective induction of the Notch ligand Jagged-1 in macrophages by soluble egg antigen from Schistosoma mansoni involves ERK signalling. Immunology.

[22] Tsao PN, Wei SC, Huang MT, Lee MC, Chou HC, Chen CY, Hsieh WS (2011). Lipopolysaccharide-induced Notch signaling activation through JNK-dependent pathway regulates inflammatory response. J Biomed Sci.

[23] Monsalve E, Perez MA, Rubio A, Ruiz-Hidalgo MJ, Baladron V, Garcia-Ramirez JJ, Gomez JC, Laborda J, Diaz-Guerra MJ. Notch-1 up-regulation and signaling following macrophage activation modulates gene expression patterns known to affect antigen-presenting capacity and cytotoxic activity. J immunol (Baltimore, Md : 1950). 2006;176(9):5362–73. 10.4049/jimmunol.176.9.5362 16622004

[24] Foldi J, Chung AY, Xu H, Zhu J, Outtz HH, Kitajewski J, Li Y, Hu X, Ivashkiv LB. Autoamplification of Notch signaling in macrophages by TLR-induced and RBP-J-dependent induction of Jagged1. J immunol (Baltimore, Md : 1950). 2010;185(9):5023–31. 10.4049/jimmunol.1001544 PMC3010732 20870935

[25] Ortiz-Masia D, Cosin-Roger J, Calatayud S, Hernandez C, Alos R, Hinojosa J, Esplugues JV, Barrachina MD (2016). M1 Macrophages activate Notch signalling in epithelial cells: relevance in Crohn's disease. J Crohns Colitis.

[26] Ryzhov S, Matafonov A, Galindo CL, Zhang Q, Tran TL, Lenihan DJ, Lenneman CG, Feoktistov I, Sawyer DB (2017). ERBB signaling attenuates proinflammatory activation of nonclassical monocytes. Am J Phys Heart Circ Phys.

[27] Wang H, Jin Y, Reddy MV, Podolsky R, Liu S, Yang P, Bode B, Reed JC, Steed RD, Anderson SW (2010). Genetically dependent ERBB3 expression modulates antigen presenting cell function and type 1 diabetes risk. PLoS One.

[28] Xu G, Watanabe T, Iso Y, Koba S, Sakai T, Nagashima M, Arita S, Hongo S, Ota H, Kobayashi Y (2009). Preventive effects of heregulin-beta1 on macrophage foam cell formation and atherosclerosis. Circ Res.

[29] Calvo M, Zhu N, Tsantoulas C, Ma Z, Grist J, Loeb JA, Bennett DL (2010). Neuregulin-ErbB signaling promotes microglial proliferation and chemotaxis contributing to microgliosis and pain after peripheral nerve injury. J Neurosci.

[30] Zhu Q, Li C, Wang K, Yue S, Jiang L, Ke M, Busuttil RW, Kupiec-Weglinski JW, Zhang F, Lu L (2017). Phosphatase and tensin homolog-beta-catenin signaling modulates regulatory T cells and inflammatory responses in mouse liver ischemia/reperfusion injury. Liver Transpl.

[31] Gopisetty A, Bhattacharya P, Haddad C, Bruno JC Jr, Vasu C, Miele L, Prabhakar BS. OX40L/Jagged1 cosignaling by GM-CSF-induced bone marrow-derived dendritic cells is required for the expansion of functional regulatory T cells. J immunol (Baltimore, Md : 1950). 2013;190(11):5516–25. 10.4049/jimmunol.1202298 PMC3660466 23630352

[32] Kuijk LM, Verstege MI, Rekers NV, Bruijns SC, Hooijberg E, Roep BO, de Gruijl TD, van Kooyk Y, Unger WW (2013). Notch controls generation and function of human effector CD8+ T cells. Blood.

[33] Higashi T, Hashimoto K, Takagi R, Mizuno Y, Okazaki Y, Tanaka Y, Matsushita S (2010). Curdlan induces DC-mediated Th17 polarization via Jagged1 activation in human dendritic cells. Allergol Int.

[34] Zheng S, Zhang P, Chen Y, Zheng S, Zheng L, Weng Z (2016). Inhibition of Notch Signaling Attenuates Schistosomiasis Hepatic Fibrosis via Blocking Macrophage M2 Polarization. PLoS One.

[35] Kibbie J, Teles RM, Wang Z, Hong P, Montoya D, Krutzik S, Lee S, Kwon O, Modlin RL, Cruz D (2016). Jagged1 instructs macrophage differentiation in leprosy. PLoS Pathog.

[36] Zhang W, Xu W, Xiong S (2010). Blockade of Notch1 signaling alleviates murine lupus via blunting macrophage activation and M2b polarization. Journal of immunology (Baltimore, Md : 1950).

[37] Antfolk D, Sjoqvist M, Cheng F, Isoniemi K, Duran CL, Rivero-Muller A, Antila C, Niemi R, Landor S, Bouten CVC (2017). Selective regulation of Notch ligands during angiogenesis is mediated by vimentin. Proc Natl Acad Sci U S A.

[38] Sonoshita M, Aoki M, Fuwa H, Aoki K, Hosogi H, Sakai Y, Hashida H, Takabayashi A, Sasaki M, Robine S (2011). Suppression of colon cancer metastasis by Aes through inhibition of Notch signaling. Cancer Cell.

[39] Rangel MC, Bertolette D, Castro NP, Klauzinska M, Cuttitta F, Salomon DS (2016). Developmental signaling pathways regulating mammary stem cells and contributing to the etiology of triple-negative breast cancer. Breast Cancer Res Treat.

[40] Acar A, Simoes BM, Clarke RB, Brennan K (2016). A role for Notch signalling in breast cancer and endocrine resistance. Stem Cells Int.

[41] Yahyanejad S, Theys J, Vooijs M (2016). Targeting Notch to overcome radiation resistance. Oncotarget.

[42] Zhang K, Wong P, Zhang L, Jacobs B, Borden EC, Aster JC, Bedogni B (2012). A Notch1-neuregulin1 autocrine signaling loop contributes to melanoma growth. Oncogene.

[43] Claus J, Patel G, Ng T, Parker PJ (2014). A role for the pseudokinase HER3 in the acquired resistance against EGFR- and HER2-directed targeted therapy. Biochem Soc Trans.

